# Engagement in a structured physical activity program and its effects upon health-related quality of life in elderly women: An observational study

**DOI:** 10.3389/fpsyg.2023.1135433

**Published:** 2023-03-22

**Authors:** Davide Maria Cammisuli, Ferdinando Franzoni, Jonathan Fusi, Giorgia Scarfò, Gianluca Castelnuovo

**Affiliations:** ^1^Department of Psychology, Catholic University, Milan, Italy; ^2^Department of Clinical and Experimental Medicine, University of Pisa, Pisa, Italy; ^3^IRCCS Istituto Auxologico Italiano, Clinical Psychology Research Laboratory, Milan, Italy

**Keywords:** elderly, female, physical activity, health-related quality of life, lifestyle

## Abstract

Aging is a complex process associated with an impairment in functional capacity and low health-related quality of life (HRQoL) due to a high frequency of chronic diseases in the elderly population. Regular physical activity (PA) may limit some physiological effects of sedentary lifestyle and increase life expectancy. An observational was conducted to measure the HRQoL among older adults living in the community, by comparing a convenience sample of elderly females practicing a structured program of PA from 2 to 3 times per week in 60-min sessions (i.e., active females, AFs) to a sample of participants adopting lifestyle behaviors expending little energy (i.e., sedentary females, SFs). A validated questionnaire (the RAND 36-item) was used as outcome measure. All participants were assessed in terms of cognitive status (Montreal Cognitive Assessment, MoCA) to exclude mild cognitive impairment, divided attention/executive functioning (Trail Making Test, Stroop Test) and psychopathological dimensions of anxiety (Geriatric Anxiety Inventory, GAI), and depression (Geriatric Depression Scale, GDS). Non-parametric analysis revealed that AFs and SFs differed significantly in the RAND Energy/Fatigue (E/F), Emotional Wellbeing (EW), and Social Functioning (SoF), that was however influenced by education level. Moreover, E/F and EW were negatively associated to anxiety and depression, while SoF was influenced by divided attention. PA results in a healthful behavior for combating feelings of fatigue and low energy as well as emotional distress that can affect health status perception in older women.

## 1. Introduction

The world population is getting older and the percentage of elderly people above 65 years will be approximately 16% in 2050 (i.e., one in 6 people; United Nations, 2022). However, this increasing trend inevitably leads to considerable social and economic impact. As the population continues to age with a parallel decline in fertility rates, regular physical activity (PA) is considered a relevant healthy behavior that can counteract physiological decline in the elderly alongside balanced diet and social engagement (Cammisuli et al., 2022). Regular PA of moderate intensity has been recognized as a significant beneficial factor for health, reducing the risk of heart disease, stroke, hypertension, type 2 diabetes, colon cancer, breast cancer, osteoporosis, and even depression and anxiety (Grande et al., 2019; Cunningham et al., 2020). In addition, PA positively affects neuronal functions, since it promotes neurotrophic factor production and synaptic plasticity as well as decreasing brain inflammation and oxidative stress (Scarfò et al., 2002a). However, a large part of the population is low-active or sedentary, engaging in levels of PA insufficient for health gain. Recently, systematic reviews with meta-analysis has shown that PA reduces the risk of early mortality (Grande et al., 2019) and may prevent falls (Sherrington et al., 2020) in the elderly.

PA is defined as any body movement produced by skeletal muscle resulting in a defined quantity of energy expenditure (Langhammer et al., 2018). PA is essential for healthy aging. According to the recommendations from the World Health Organization (2010), adults aged 65 and older should perform at least 150 min per week of moderate intensity activity (e.g., brisk walking) or 75 min a week of vigorous intensity activity (e.g., hiking, jogging, or running). It has been recommended that a program of regular exercise including cardiorespiratory, resistance, flexibility, and neuromotor training can improve physical fitness and is essential for health status vitality in most adults (Garber et al., 2011; Rivera-Torres et al., 2019). Different exercise interventions can improve functional capacity in frail subjects like older adults. Particularly, multicomponent exercise programs involving strength, endurance, balance and flexibility activities may be more effective especially when undertaken regularly (Losa-Reyna et al., 2019). For optimal effects, older adults have to adhere to structured exercise programs near the maximum capacity to stimulate body systems and induce improvements in physiological parameters, such as the VO2max and muscular strength (Langhammer et al., 2018). A previous meta-analysis (Daskalopoulou et al., 2017) showed that a diminished risk of functional limitation and disability is observed in older people attending regular physical exercise. It is also well-established that lifestyles including PA in the elderly contribute to better executive functioning, as higher level cognitive processes that are fundamental in targeting goals, effortful behavior and environment adaptation (Corbo and Casagrande, 2022). Elderly people preserve some cognitive skills (e.g., language and crystallized intelligence as knowledge of general facts) while other cognitive processes like executive functions deteriorate and interfere with daily routines (Casagrande et al., 2021). To counteract this trend, PA programs are well-established strategies for improving working memory, cognitive flexibility, and inhibitory control within the realm of executive functions in cognitively healthy older adults, as recently reported in a systematic review with meta-analysis of randomized controlled trials (Xiong et al., 2021).

Older adults are among the most physically inactive part of the society. Aging is a multifactorial irreversible process both associated with neuromuscular functions decline and health-related quality of life (HrQoL) decrement. The HrQoL relates specifically to a person’s health and refers to a measure of a person’s functioning, wellbeing and general health perception in each of the three domains: physical, psychological and social (Apolone and Mosconi, 1998). Functional mobility supported by PA programs and cognitive vitality are among the pivotal factors for independence in everyday life of elderly people (Demnitz et al., 2018). Measures of HrQoL are commonly used to determine the effect of medical treatments or to conduct community-based surveys, to compare different clinical populations and assess needs of older adults as potential beneficiaries of public health interventions (Walters et al., 2001). An Italian survey has recently confirmed that higher prevalence of health status negative perception is associated with older age and this negative impression is more frequent in women than in man (Giacomozzi et al., 2020). Moreover, self-report health is associated with poor mental health and in recent years a growing body of evidence has reported the relation between negative aging perception and anxiety/depression (Freeman et al., 2016).

Literature shows a paucity of research about PA benefits for psychological wellbeing in elderly females, given that women are usually less engaged than men in practicing regular exercise (*cf*., Pruneti et al., 2019). Moreover, there is an urgent need for global policy on physical inactivity, since one in four adults are insufficiently active with higher rates in women than men (World Health Organization, 2018). Thus, more investigation in this area can inform the direction of future intervention programs. In fact, PA is widely recognized as relevant for supporting healthy aging in different ways, specifically as a key enabler of social participation, personal autonomy, dignity, and greater psychological wellbeing and quality of life (World Health Organization, 2018). After the development of “ACTIVE,” a technical toolkit created to support countries in increasing PA levels and providing a roadmap for improving health and wellbeing according to the Global Action Plan 2018–2030, the WHO commissioned a scoping review (Taylor et al., 2021) to assess the available volume of evidence in this area and inform the direction of further research. As a result, this review found that only few studies have targeted outcomes of social functioning as well as wellbeing/quality of life in relation to PA in the elderly.

PA is a key element of the “*Healthy Aging*” defined as the “*process of developing and maintaining the functional ability that enables wellbeing in older age*” (World Health Organization, 2019).^1^ In fact, maintaining healthy behaviors throughout life, particularly regular PA, contribute to reducing the risk of chronic diseases, improving motor skills and mental abilities and delaying caretakers’ dependency. To date, research on HrQoL and PA has rarely investigated how this relationship differ in relation to physical exercise levels in older women. In the light of this assumption, we hypothesize that older women practicing PA regularly have a better perception of health status and show increased mental abilities than sedentary peers. The present study aimed at verifying differences in health-related quality of life (HrQoL) perception as a key dimension of psychological wellbeing of elderly people and in frontal domains efficiency, by comparing a group of active versus sedentary community-dwelling older women. Particularly, we expect that active females better perceive specific aspects of their health status, and show improved executive functioning than sedentary females.

## 2. Method

### 2.1. Participants

A total of 25 elderly females (mean age, 74.04 ± 3.84) constituted the sample of the study. Inclusion criteria encompassed the following ones: age ≥ 65 years; absence of motor impairment able to prevent physical exercise. Participants were excluded if they report neurological or psychiatric diseases not compatible with PA or medical illnesses that may get worse by physical exercise and if they report established cognitive difficulties that make them unable to reply to neuropsychological tests and complete psychological questionnaires or present with visual impairment. A convenience sample of community-dwelling older females was recruited in the city of Lucca (Tuscany, Italy). The sample was then divided into two groups, according to PA level: active females (AFs) (*n* = 15) following a structured multidimensional protocol (SMP) for elderly people at the community gym (2–3 times a week in 60-min sessions), and sedentary females (SFs) (*n* = 10) only characterized by little expenditure of physical effort during routinely activities of daily living at home and not involving in the SMP proposed by the community gym. Participation in the study was voluntary and individuals were able to withdraw at any point without providing a reason. All participants signed the informed consent before starting the evaluation and the research was conducted under the principles of the Declaration of Helsinki. The study was approved by the Bioethical Committee of Pisa.

### 2.2. SMP of PA in the AF group

The AFs group had been participating the SMP of PA at the community gym for 6 months. Each 60-min training session of PA was divided into six parts: (1) a 10-min warm-up, with walking and mobility exercises of the neck, wrist, scapulohumeral, spine, hip and ankle joints; (2) a 10-min play-coordination part, consisting of exercises with the ball which include relay races, slalom, bowling, and basket shooting; (3) a static and/or dynamic stretching of the main muscle groups/chains; (4) a central part lasting about 20 min with balance exercises, motor control, muscle strengthening with the help of the back, dumbbells and TRX (this part includes functional and multi-joint exercises such as squats, horizontal pull-ups, vertical and horizontal thrusts, back lunges, hip extensor exercises, and quadriceps strengthening exercises); (5) an intermittent aerobic exercise using the Step, a simplified version of Jumping Jacks, or brisk walking movements alternating with slow walking for about 10 min; (6) relaxation exercises through the use of postures and breathing for about 10 min.

### 2.3. Assessment

#### 2.3.1. Health-related quality of life evaluation

The RAND 36-item Health Survey 1.0 (Hays et al., 1993) is a multi-purpose tool designed to detect adults’ perceptions about their own health. It consists of 36 items identical to the MOS SF-36 described in Ware and Sherbourne (1992). They were adapted from longer instruments involved in the Medical Outcomes Study (MOS) (Hays and Shapiro, 1992). It encompasses eight health dimensions: Physical Functioning (PF); Bodily Pain (BP); Role limitations due to physical health problems (RP); Role limitations due to emotional problems (RE); Emotional wellbeing (EW); Social Functioning (SoF); Energy/Fatigue (E/F); General Health perception (GH). The survey also includes a single item providing an indication of perceived change in health (HC). The RAND-36 takes about 7–10 min to self-administer. The most important effort to produce equivalent translations for use across linguistic groups of the RAND-36 (aka the SF-36) was the International Quality of Life Assessment (IQOLA) study (Aaronson et al., 1992). Each domain is scaled from 0 to 100, with higher scores indicating satisfaction in perceived HrQoL. An IQOLA project investigation on SF-36 has confirmed the internal consistency reliability of dimensions (from 0.68 to 0.94; Alonso et al., 2004). General practitioners services support the construct validity of this instrument for the over −65 s (Lyons et al., 1994; Walters et al., 2001).

#### 2.3.2. Psychological measures

The levels of anxiety and depression experienced by the patients were assessed by the Geriatric Anxiety Inventory (GAI; Ferrari et al., 2017) and by the Geriatric Depression Scale (GDS; Yesavage et al., 1982), respectively. The GAI consists of 20 items, is self-administered and takes a short compilation time of about 5 min. It specifically evaluates anxiety in the elderly, including potential somatic symptoms experienced by older adults pertaining to how the individual felt in the past week. The Italian validation (Ferrari et al., 2017) confirmed a good reliability, with a Cronbach’s alpha of 0.77. The cut-off to identify anxiety symptoms is fixed at 8/9 points in the non-clinical population. The GDS represents the most specific psychodiagnostic test built on the characteristics of depression in old age. The instrument has been extensively used in community, acute care and long-term care settings. It is a self-report scale developed in 30 items in the original version. The computed value of the alpha coefficient in the original study was 0.94, suggesting a high degree of internal consistency for the GDS. The cut-offs for normal status, mild depression and severe depression occur at 0–9 points, 10–19 points, and 20–30 points, respectively.

#### 2.3.3. Global screening measure

MoCA (Nasreddine et al., 2005). It is a neuropsychological test used as a screening tool for cognitive deterioration, particularly sensitive in the case of Mild Cognitive Impairment. It consists of 12 sub-tasks including: verbal memory (assessed by the delayed recall of five nouns); visual constructive skills (assessed by a clock-drawing task and a copy of a cube); executive functions (assessed by a brief version of the Trail Making Test, a phonemic fluency task, and a verbal abstraction task); attention, concentration, and working memory (assessed by means of a sustained attention task, a serial subtraction task, a forward and backward span tasks for digits); language (assessed by a naming task of low familiarity animals, repetition of two syntactically complex sentences and the abovementioned phonemic fluency task); temporal and spatial orientation (year, month, day of the week, exact date, place, location). In the original study, test/retest reliability was high (*r* = 0.92, *p* < 0.001) and internal consistency was good (Cronbach’s alpha = 0.83). The maximum total score is 30 points. Raw scores were transformed into correct scores for age and education for total score and sub-scores of visual constructive skills, executive functions, language and orientation and also for gender in relation to attention, concentration and working memory. A correct score ≤ 15.5 constitutes the pathological cut-off calculated for the Italian calibration (Santangelo et al., 2015) that was adopted for the present study.

#### 2.3.4. Attention and executive measures

The Stroop test is a neuropsychological tool widely used to assess selective attention, cognitive flexibility and sensitivity to interference related to frontal lobe efficiency. The test consists of three trials. In the first trial, the subject is required to read three lists of color names (i.e., green, red, blue) in a random order as fast as he/she can. In the second trial, the subject is required to read three lists of colored dots (i.e., green, red, blue) in a random order as fast as he/she can. In the third trial, the subject is required to read three lists of words written in a different color (e.g., the word “RED” written in blue ink) in all possible combinations and in a random order. In this incongruent condition, the examinee is required to name the color of the ink instead of reading the word. The average execution time in seconds of each subtest is 14.9, 20.7, and 40.8 s, respectively. The last trial measures sensitivity to interference (i.e., the ability to inhibit an automatic response). The test returns two scores: sensitivity to interference related to the time spent in completing the trials (i.e., Interference/Time) and sensitivity to interference related to committed errors (i.e., Interference/Errors). Raw scores (i.e., performances in seconds) were transformed into correct scores for age and education. The test has good psychometric properties (Brugnolo et al., 2016). Normative data for the Italian population were also provided by Caffarra et al. (2002) on a shortened version of the test developed by Venneri et al. (1993), which is widely used in the assessment of patients with dementia. Correct scores ≥4.25 and ≥ 36.92 constitute the pathological cut-offs for Interference/Error and Interference/Time, respectively.

The Trail Making Test (TMT) is used to assess visual processing speed and divided attention/attention shifting. The test is composed of two separate tasks (i.e., A and B). In Part A, the examinee is required to connect 25 numbered circles by a pen with a direct line according to the ascending order of numbers (i.e., 1–2–3, and so on). Time needed to complete Part A (in seconds) is considered a measure of visual processing speed. In Part B, the examinee is required to connect numbered and lettered circles respecting the alternating sequence of numbers and letters, that is the alphabetical and numerical series in progressive order (i.e., 1-A, 2-B, 3-C, and so on). Time needed to complete Part B (in seconds) is considered a measure of divided attention. TMT B-A score (in seconds) is considered the pure measure of divided attention net of visual processing speed. This derived score is usually used to remove the speed component from the performance on the test, providing a refined estimation of executive efficiency related to prefrontal activation (Arbuthnott and Frank, 2000). Basic scores and derived TMT B-A score showed a good test–retest reliability (Part A, *r* = 0.80; Part B, *r* = 0.81, Part B-A, *r* = 0.70). The raw scores of Part A (i.e., TMT-A score), Part B (i.e., TMT-B score) and TMT B-A are converted into correct scores for age and education. Scores equal or greater than 127 s. for Part A, 294 s. for Part B and 163 s. for B-A are considered pathological. The calibration of the test used for this study (Siciliano et al., 2019) contemplates norms for older adults (age-ranges: 60–69, 70–79, 80–89).

The Visual Search Test evaluates selective attention and consists of 3 different arrays of numbers. The examinee has to cross out the target numbers which are one for the first matrix (number 5), two for the second matrix (number 2 and number 6), and three for the third matrix (number 1, number 4 and number 9). The target stimuli are 10 in the first matrix, 20 in the second matrix and 30 in the third matrix. The first line of each matrix serves as an example and is filled by the examiner. The second line acts as a run-in and is filled by the examinee. The compilation time is recorded by means of the stopwatch. The stimuli crossed out within 45 s of time for each matrix are counted. Errors should not be corrected. The administration time is approximately 5 min. Psychometric properties are reported in Spinnler and Tognoni’s (1987) review of Italian neuropsychological tests.

### 2.4. Statistical analysis

A single-center observational study was performed. The normality of the collected data was tested by the Shapiro–Wilk. In order to control for potential influence of socio-demographic parameters, global cognitive status and psychological variables on health status perception, we first compared the two groups. To this end, independent samples *T*-tests were used for normally distributed data and Mann–Whitney U-tests were used for non-Gaussian distributions.

A non-parametric analysis (i.e., the Mann–Whitney *U*-test, Bonferroni corrected) was then applied to compare groups’ performances on HrQoL and neuropsychological measures. A Spearman-Rank correlation was used to further investigate the association between HrQoL dimensions and neuropsychological and clinical variables, too. All analyses were run by the Statistical Package for the Social Sciences (SPSS) software for Windows (SPSS, version 23.0; SPSS, Inc., Chicago, IL).

## 3. Results

Results of the comparison between groups were shown in Table 1. Variables were expressed as median values except for age, MoCA total score, GDS and GAI expressed as mean ± *standard deviation*.

**Table 1:** Results of the comparison between groups.

	AF	SF	*p-*value
Age	74.26 ± 4.26	73.70 ± 3.30	n.s
Education	13.00	6.50	*p* = **0.019**
MoCa total score	24.48 ± 2.84	24.32 ± 1.89	n.s
MoCa visuospatial	3.73	3.42	n.s
MoCA executive	3.52	3.79	n.s
MoCa language	5.63	5.99	n.s
MoCa orientation	6.06	6.14	n.s
MoCa attention	5.09	5.87	n.s
MoCa memory	3.00	2.00	n.s
GDS	6.73 ± 4.33	8.70 ± 4.19	n.s
GAI	7.07 ± 5.28	7.60 ± 6.27	n.s.
Visual search	55.25	54.00	n.s
TMT A	40.00	51.00	n.s
TMT B	73.00	49.00	n.s
TMT B-A	29.00	−5.000	n.s
Stroop interference/Time	11.10	15.25	n.s
Stroop interference/Error	−0.75	−1.20	n.s
RAND PF	85.00	72.50	n.s
RAND RF	75.00	25.00	n.s
RAND RE	100.00	33.35	n.s
RAND E/F	60.00	50.00	*p* = **0.019**
RAND E/W	68.00	54.00	*p* = **0.023**
RAND SoF	75.00	50.00	*p* = **0.014**
RAND BP	55.00	45.00	n.s
RAND GH	60.00	45.00	n.s
RAND HC	50.00	50.00	n.s

MoCA, Montreal Cognitive Assessment; GDS, Getriatric Depression Scale; GAI, Geriatric Anxiety Inventory; TMT, Trail Making Test; PF, Physical Functioning; BD, Bodily Pain; RP, Role limitations due to physical health problems; RE, Role limitations due to emotional problems; EW, Emotional wellbeing; SF, Social Functioning; E/F, Energy/Fatigue; GH, General Health; HC, perceived change in health. Significant results are in bold.

The groups were matched according to age (*t* = 0.354, *gl* = 23, *p* = 0.726), level of depression (*t* = −1.126, *gl* = 23, *p* = 0.272), anxiety (*t* = −0.229, *gl* = 23, *p* = 0.821), and global cognition (*t* = 0.148, *gl* = 22, *p* = 0.883), but not in terms of education (*U* = 33.000, *Z* = −2.368, *p* = 0.019).

No significant comparisons on attention and executive measures were revealed between groups. Conversely, AFs showed a greater health status perception in terms of RAND E/F (*p* = 0.19, *η*^2^ = 0.23), EW (*p* = 0.023, *η*^2^ = 0.21), and SoF (*p* = 0.014, *η*^2^ = 0.26) than SFs. However, education was positively associated with SoF (rho = 0.508, *p* < 0.01). No other significant correlations were found between education and the other RAND dimensions.

The RAND E/F and EW sub-scores were negatively associated with the GAI scores (rho = −0.464 and rho = −0.474, respectively) (*p* < 0.05). The RAND EW, RP, and RE sub-scores were negatively associated with the GDS scores (rho = −0.435, rho = −0.582, and rho = −0.400, respectively) (*p* < 0.05), too. The RAND SoF was positively associated with the TMT B-A scores (rho = 0.501, *p* < 0.05), the RAND RP was positively associated with TMT Part B scores (rho = 0,450, *p* < 0.05), and RAND HC was positively associated with TMT B-A scores (rho = 0,521, *p* < 0.01). No other significant correlation was found between the RAND sub-scores and neuropsychological measures (Table 2).

**Table 2:** Spearman’s rho coefficients between RAND dimensions and psychological and neuropsychological measures.

	GDS	GAI	Stroop Int./Time	Stroop Int./Error	TMT Part A	TMT Part B	TMT B-A
RAND PF	−0.314	−0.056	0.064	−0.092	0.020	0.196	0.288
RAND BP	−0.282	−0.245	0.070	0.161	−0.368	0.214	0.230
RAND RP	−0.582*	−0.329	−0.168	0.061	0.084	0.450*	0.364
RAND RE	−0.400*	−0.211	−0.178	0.066	−0.054	0.327	0.302
RAND EW	−0.435*	−0.474*	−0.090	−0.147	0.072	0.344	0.278
RAND SoF	−0.270	−0.080	−0.175	−0.159	−0.052	0.241	0.501*
RAND E/F	−0.365	−0.464*	−0.009	−0.155	−0.018	0.171	0.177
RAND GH	−0.213	−0.047	0.052	−0174	−0.081	0.290	0.287
RAND HC	−0.275	−0.129	−0.269	−0.178	−0.399	0.309	0.521**

RAND PF, Physical Functioning; RAND RP, Bodily Pain (BP); RAND RP, Role limitations due to physical health problems; RAND RE, Role limitations due to emotional problems; RAND EW, Emotional wellbeing; RAND SoF, Social Functioning; RAND E/F, Energy/Fatigue; RAND GH, General Health; RAND HC, Perceived change in health; GDS, Geriatric Depression Scale; GAI, Geriatric Anxiety Scale; Stroop Int./Time, Stroop Interference/Time; Stroop Int./Error, Stroop Interference Error; TMT Part A, Trail Making Test, Part A; TMT Part B, Trail Making Test, Part B; Trail Making Test B-A, Trail Making Test, Part B-Part A. *p < 0.05;**p < 0.01.

## 4. Discussion

This informative brief report focused on filling information gap presented in literature regarding HrQoL perception in active and sedentary older women living in the community. Our hypothesis was partially corroborated by the study results reporting that AFs practicing a SMP of PA (i.e., 60-min sessions for 2/3 times a week) better judge their health status for specific aspects, such as perceived levels of physical/mental energy (RAND E/F) and emotional wellbeing (EW) than SFs not involved in any structured PA, although the effect size was quite low. The association between education and RAND SoF prevent us to directly conclude in favor of a third significant difference among groups, given that the former variable somehow influences the latter variable in the AFs group presenting a higher level of schooling. Conversely, our hypothesis was disconfirmed by the results whit regard to attention and executive measures that were not higher in the AFs group than SFs group.

In more detail, findings on RAND E/F corroborate that physical health declines steeply with age, especially in SFs. Fatigability represents a valid measure of PA-related energy expenditure in older adults (Buchowski et al., 2013). Fatigue is one of the most common reported symptoms in primary care settings by older adults experienced as distressing and as a limit for social participation (Egerton et al., 2016). Health perception in older adults is substantially influenced by physical feelings of fatigue (Vetrovsky et al., 2021) that are reduced in those practicing PA.

Further, research has pointed out that engagement in structured PA protocols can improve some aspects of psychological wellbeing (RAND EW), such as mood and self-perception in the elderly (Fox et al., 2007). Additionally, RAND EW was negatively associated with anxiety (GAI scores) and depression levels (GDS scores), as expected (Maldonado Briegas et al., 2020). In this regard, it is well known that depressive states and any other psychological alteration like anxious symptoms are associated with worse metabolic profile that negatively reflects on health status way of viewing, so PA becomes fundamental in preventing psychiatric disorders (Scarfò et al., 2022b), too. Moreover, lower depression levels (GDS scores) correspond to minor limitations due to emotional (RAND RE) or physical problems (RAND RP), as expected, given the widely accepted capacity of physical exercise in exerting positive effects on mood for older adults (Miller et al., 2019).

Lastly, HrQoL – in terms of social functioning (RAND SoF) and especially as globally perceived in the last year (RAND HC) – was positively correlated to divided attention (TMT B-A), that can be defined as the neurocognitive ability to simultaneously attend to more than one task. This ability is crucial for a number of everyday duties, such as walking or driving which are fundamental for older adults to maintain their independence from caregivers (Fraser and Bherer, 2013). Further, a diminished divided attention in the elderly may be due to physical problems (RAND RP).

The absence of any effects of PA on attention and executive measures in the AFs could be explained by some methodological restraints of our report, such as the absence of a probabilistic sampling with low size as well as study design. The impact of structured PA programs on frontal domains efficiency should be investigated by randomized intervention trials with follow-ups for testing maintenance effects. However, our observational study was useful in starting to probe HrQoL in active and sedentary older females. We would like to stress that a comparison with a group of males would also clarify which health dimensions may benefit most from PA in relation to gender. With regard to the assessment, a larger neuropsychological tests battery including the evaluation of the memory system should be also used in future investigations, in order to evaluate a key dimension of cognitive decline in the elderly. Another limitation of our investigation is represented by the fact that we did not use any instrument to detect comorbidity (e.g., the Cumulative Illness Rating Scale, CIRS) able to quantify health-related problems in the geriatric population. In our opinion, future studies should include clinical variables (e.g., BMI) and motor performances (e.g., Time Up and Go Tests, 6-min walking test, 10-m walking test, etc.) as well as measures for assessing how close a diet is to a healthy nutrition (e.g., the Mediterranean Adequacy Index of Italian diets; Alberti-Fidanza and Fidanza, 2004), and questionnaires evaluating activity engagement in old age and cognitive reserve (e.g., The Cognitive Reserve Questionnaire, CRQq; Nucci et al., 2012), with the final aim of evaluating other relevant variables that may influence health status perception beyond PA protocol attendance in cognitively healthy older women.

## 5. Conclusion

According to the last WHO guidelines on physical activity and sedentary behavior (World Health Organization, 2020a,b), our study documented that engaging in a multicomponent structured program of PA 2/3 times per week in 60-min sessions targeting functional balance, cardiorespiratory fitness and muscle strength promotes the maintenance of physical/mental energy with reduced fatigue levels and emotional wellbeing, with positive implications for health status perception in elderly females. That is also strongly related to decreased anxiety whereas depression seems to be further influenced by females’ perception limitations due to emotional and physical problems of the elderly. Finally, self-perception of social functioning and especially perceived health changes call into question the cognitive ability of attentional shifting, as a core domain regulating dual-task performances of relevant daily living activities in older people for their independence, that can be affected by limitations due to psychical problems.

## Data availability statement

The datasets presented in this article are not readily available because the dataset is restricted to the authors’ usage. Requests to access the datasets should be directed to ferdinando.franzoni@unipi.it.

## Ethics statement

The studies involving human participants were reviewed and approved by the Bioethical Committee of Pisa. The patients/participants provided their written informed consent to participate in this study.

## Author contributions

DC and FF: methodology and writing-original draft preparation. DC, FF, and JF: data curation. GS and JF: writing-review and editing. GC: visualization, supervision, and project administration. All authors contributed to the article and approved the submitted version.

## Funding

This research received funding from the Italian Ministry of Health.

## Conflict of interest

The authors declare that the research was conducted in the absence of any commercial or financial relationships that could be construed as a potential conflict of interest.

## Publisher’s note

All claims expressed in this article are solely those of the authors and do not necessarily represent those of their affiliated organizations, or those of the publisher, the editors and the reviewers. Any product that may be evaluated in this article, or claim that may be made by its manufacturer, is not guaranteed or endorsed by the publisher.

## Footnotes

^1^ https://www.who.int/docs/default-source/documents/decade-of-health-ageing/decade-healthy-ageing-update-march-2019.pdf?sfvrsn=5a6d0e5c_2#:~:text=Healthy%20Ageing%20is%20the%20process,enables%20wellbeing%20in%20older%20age
