# Corrigendum: Engagement in a structured physical activity program and its effects upon health-related quality of life in elderly women: an observational study

**DOI:** 10.3389/fpsyg.2023.1298200

**Published:** 2023-11-07

**Authors:** Davide Maria Cammisuli, Ferdinando Franzoni, Jonathan Fusi, Giorgia Scarfò, Gianluca Castelnuovo

**Affiliations:** ^1^Department of Psychology, Catholic University, Milan, Italy; ^2^Department of Clinical and Experimental Medicine, University of Pisa, Pisa, Italy; ^3^IRCCS Istituto Auxologico Italiano, Clinical Psychology Research Laboratory, Milan, Italy

**Keywords:** elderly, female, physical activity, health-related quality of life, lifestyle

In the original article, there was an error in affiliation 3 as published. Instead of “Psychology Research Laboratory, Istituto Auxologico Italiano, Milan, Italy”, it should be “IRCCS Istituto Auxologico Italiano, Clinical Psychology Research Laboratory, Milan, Italy”.

The authors apologize for this error and state that this does not change the scientific conclusions of the article in any way. The original article has been updated.

